# Survey of the intersection of mental maps and visualization

**DOI:** 10.1186/s42492-026-00225-1

**Published:** 2026-07-15

**Authors:** Jie Guo, Huiyuan Shi, Yucheng Kong, Jiazhen Du, Zeyang Liu, Fangfang Zhou, Ying Zhao

**Affiliations:** School of Computer Science and Engineering, Central South University, Changsha 410083, Hunan China

**Keywords:** Mental maps, Visualization, Interdisciplinary field, Survey

## Abstract

The intersection between mental maps and visualization has attracted increasing research attention in recent years, as they exhibit a natural functional complementarity. Mental maps research explores how humans construct in-mind representations of the external world through the view of cognitive science, while visualization can transform implicit mental maps into concrete visual forms, thereby facilitating the analysis of their construction mechanisms. Visualization, a branch of computer science, investigates how to design human-friendly visual representations of digital data. Mental maps can provide human-perspective insights to guide visualization designs and improve the effectiveness of data understanding and analysis. This paper presents a literature survey of the recent research at this intersection. Regarding the visualizations of mental maps, the present study classifies relevant research into visualizations to depict structural topographies, attribute information in mental maps, and compare multiple mental maps. In terms of mental maps for visualization, this study categorizes the relevant research into the element structuring, structural mapping, and consistency maintenance mechanisms of mental maps to guide visualization designs. This paper introduces and discusses in detail representative works in these categories, providing future research opportunities at this intersection.

## Introduction

Mental maps, which originated from cognitive psychology and geography [1–4], refer to the internal and structural representations of the external world that humans construct in their minds based on perception, memory, and experience [1, 5, 6]. The construction of mental maps supports daily human behaviors, such as positioning, navigating, and complex decision-making. Research on mental maps mainly focuses on exploring their construction mechanisms and analyzing the principles of how to support human daily behaviors. Visualization originated from computer graphics [7, 8] and human-computer interaction (HCI) [9–11], and refers to the processes of transforming abstract data into visual representations [12–15]. These processes assist humans in understanding and utilizing in-depth data. Research on visualization focuses on how to best map data to visual representations for data analysis.

Mental maps and visualization present a natural complementarity in the function [5, 16]. Visualization can transform abstract mental maps into concrete forms to facilitate the analysis of the construction mechanisms and working principles of mental maps. Mental maps provide human-perspective insights into the design of data mapping, thereby enhancing the efficacy of visual representations in supporting data analysis. This complementarity provides a solid basis for advancing interdisciplinary research [17–20]. Numerous studies have focused on the intersection of mental maps and visualization [21, 22]. Although some surveys have discussed spatial cognition, mental maps, and visualization design, only limited attention has been paid to the interdisciplinary intersection between mental maps and visualization.

Thus, the present study aims to provide a systematic survey of existing studies on the intersection of mental maps and visualization. To identify relevant studies, we searched Google Scholar for papers published between 2000 and 2025 using combinations of terms related to mental maps, spatial cognition, and visualizations, using “mental maps” as the primary term and “cognitive maps” as a closely related concept. When existing studies used the term “cognitive maps” we determined whether they fell within the scope of this survey according to whether they investigated internal representations of the external world and their relationships with visualization. Duplicate papers, papers without accessible full texts, and papers that only discussed mental maps or visualizations without explicitly addressing their intersection were excluded. After screening titles and abstracts and further examining the remaining papers through a full-text review, 98 research papers were selected for this survey. These papers were predominantly drawn from leading academic journals and conferences in the fields of psychology, geography, visualization, and HCI, such as Cognition, Frontiers in Psychology, The Cartographic Journal, IEEE Transactions on Visualization and Computer Graphics, and the IEEE Visualization Conference and ACM Conference on Human Factors in Computing Systems.

Drawing on a thorough literature review, the present study proposes a taxonomy for the intersection of mental maps and visualization. This paper begins with the complementarity between mental maps and visualization, classifying existing studies into two categories: visualization for mental maps and mental maps for visualization. In the category of visualization for mental maps, we further divide the relevant studies into three subcategories according to the intended purposes of the visualizations, including depicting structural information or attribute information contained in mental maps, as well as information comparisons across mental maps. Visualization approaches for transforming mental maps into visual representations are then outlined, and representative examples for each subcategory are introduced. In the category of mental maps for visualization, we further divide the relevant studies into three subcategories according to the working mechanisms of mental maps, elaborated on the specific ways in which mental maps guide visualization design, and illustrated representative examples for each subcategory. Finally, future research opportunities and challenges regarding the intersection of mental maps and visualization are discussed. Overall, the value of this survey lies in its focus on this interdisciplinary intersection and its taxonomy for systematically organizing existing studies.

## Visualization for mental maps

The visualization of mental maps aims to transform mental maps in human minds into visual representations presented on digital displays. Such visual representations predominantly present two types of information contained within mental maps, namely structural information (i.e., elements within mental maps and their relationships, such as spatial relations and path hierarchies) and attribute information (i.e., subjective properties assigned by individuals to elements within mental maps, such as emotions and attitudes). These visual representations further facilitate information comparison across different mental maps. Therefore, we classified existing studies into three categories, visualization for: (1) depicting structural information in mental maps, (2) representing attribute information in mental maps, and (3) comparing mental maps. These categories are not mutually exclusive, as some studies may adopt integrated approaches involving multiple types of information. For example, a visualization may present movement trajectories along with emotional responses, thereby involving both structural and attribute information in mental maps. In the present survey, such studies were classified according to their primary analytical purpose, namely whether they focused on structural information, attribute information, or comparisons across different mental maps. Table 1 presents a summary of the references and representatives from these three categories. The following subsections introduce the definitions and characteristics of the three categories of existing studies, and discuss how they explore the construction mechanisms and working principles of mental maps through visualization.

**Table 1:** Relevant studies on visualization for mental maps

Category	Reference	Representative
Visualization for depicting structural information in mental maps	[23–39]	[23, 27, 28, 30, 31, 35, 36]
Visualization for representing attribute information in mental maps	[40–47]	[42–44, 46]
Visualization for comparing mental maps	[48–57]	[48, 51, 56, 57]

### Visualization for depicting structural information in mental maps

Visualizations depicting structural information in mental maps aim to present the elements within mental maps, as well as the relationships among these elements. For example, in a mental map of city roads, three main roads and ten side streets form a hierarchical path structure, with the main roads serving as the backbone. Another example is a mental map for an exhibition hall layout, which includes an entrance hall and two exhibition rooms, forming a spatial structure of left-right symmetry with the entrance hall at the center and the two exhibition rooms on either side. These structures reflect how mental maps organize information regarding the external world. As these structures are implicit, visualizing them first requires obtaining data that contains structural information in mental maps.

Two types of data–drawn sketches and trajectories–are commonly adopted in existing studies to explore the structural information in mental maps. Drawn sketches refer to schematic diagrams obtained through the manual drawing of the memories and understanding of individuals of objective objects in the real world, such as the spatial structure of a campus or city [23–27]. Trajectories involve sequential records of spatial positions of individuals during spatial activities in both real and virtual environments [31–34]. Various digital and wearable devices support trajectory collection.

**Fig. 1:**
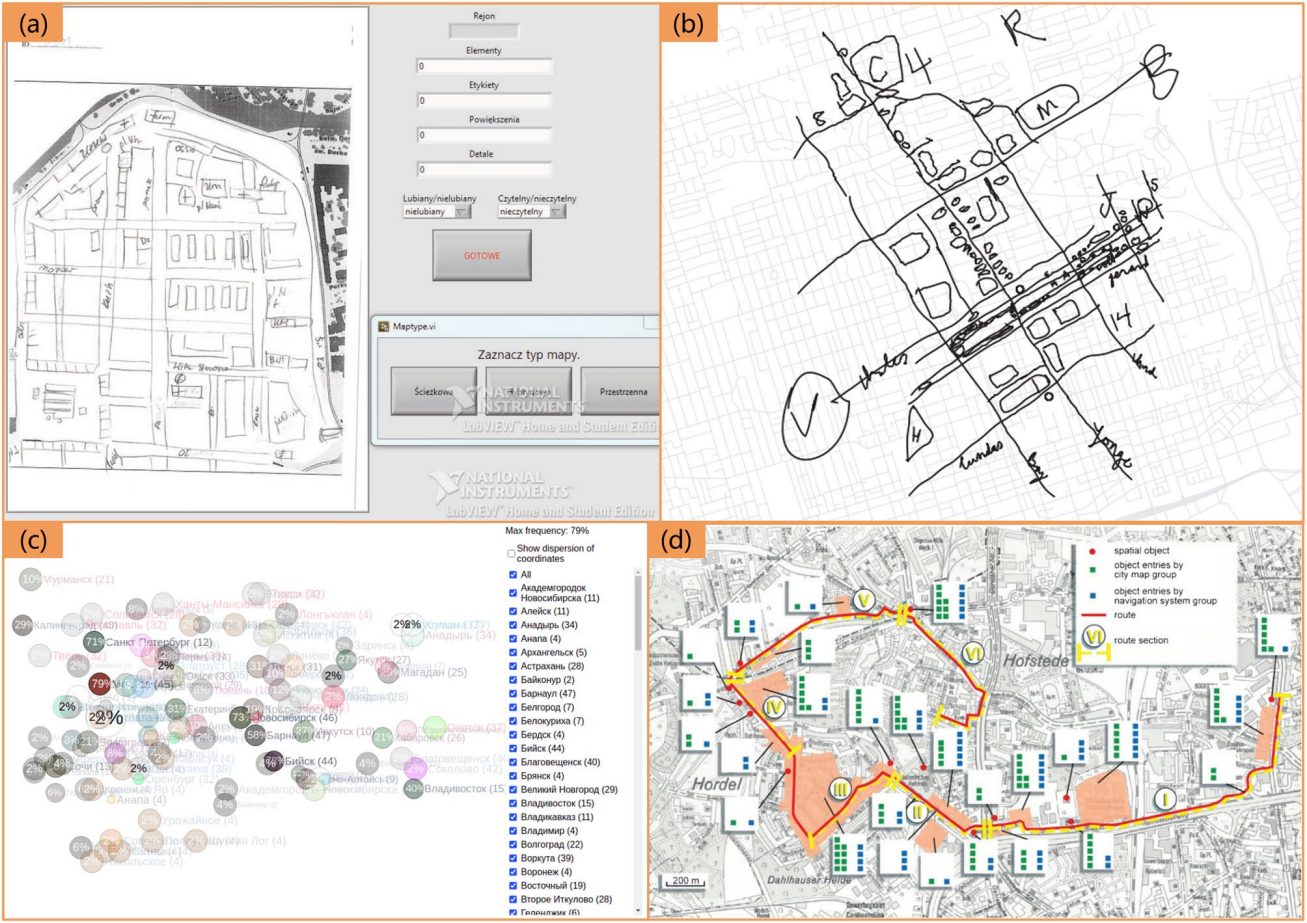
Examples of depictions of structural information in mental maps via drawn sketches. **a** A system for assisting in the identification of structural items [23]; **b** A method of layering a drawn sketch onto a map to analyze the hierarchical path structure of a city [30]; **c** A node-link graph presenting the digital data items [27]; **d** A spatial visual representation illustrating the digitized roads and landmarks contained within the drawn sketches [28]

Drawn sketches, as an intuitive form of visual representation, allow human viewers to directly observe structural items, such as roads, landmarks, and boundaries, and to analyze their interrelationships. For example, Stryjewsja et al. [23] developed a CogMap Analyst System (Fig. 1a) that helps users to easily identify roads and landmarks contained within a drawn sketch. Tu Huynh et al. [30] further layered a drawn sketch onto a map (Fig. 1b) to analyze the hierarchical pathway structure of a city formed on a mental map, namely, which roads formed the backbone and branches of the city and where the landmarks and boundaries of the city were located. Some scholars digitized sketches to facilitate the extraction and analysis of structural information formed in mental maps. For example, Chumakov et al. [27] transferred manually identified landmarks in a drawn sketch and their connected paths into digital data items and subsequently presented them as a node-link graph (Fig. 1c), which intuitively illustrates the organizational landmark-path structure formed in a mental map. Dickmann [28] further designed a spatial visual representation (Fig. 1d) to present digitized roads and landmarks contained in drawn sketches, thereby intuitively revealing the backbone roads and important landmarks underlying mental maps.

**Fig. 2:**
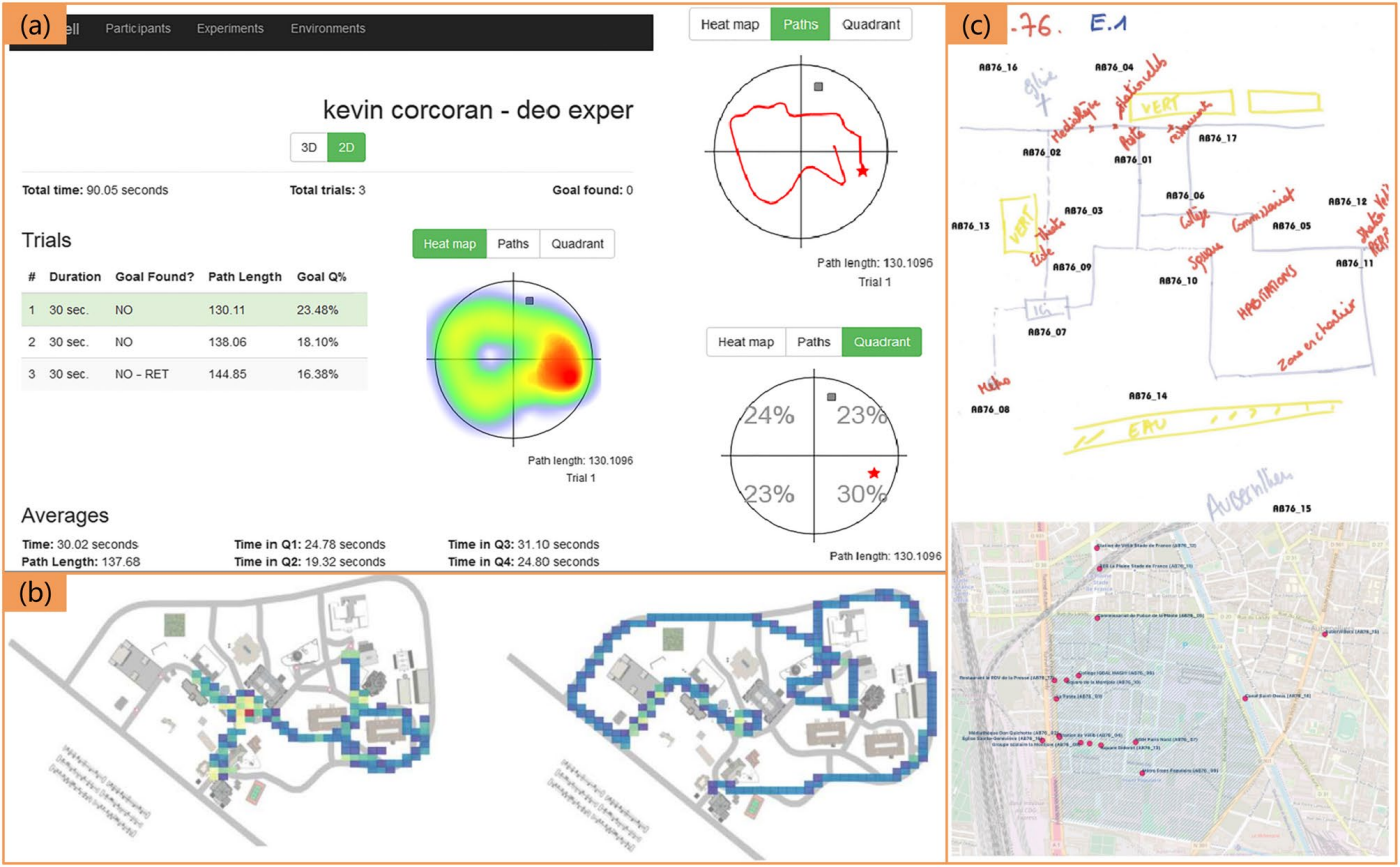
Examples of depictions of structural information in mental maps via trajectories. **a** A system that uses multiple diagrams to present the path distributions, stop regions, and dwell times within trajectories [35]; **b** Path distribution diagrams used to display movement densities in trajectories [36]; **c** Spatial distribution diagrams combined with drawn sketches to analyze the formation mechanisms of structural items within mental maps [31]

Trajectories are inherently digitized and contain spatial positions as fundamental information and various spatiotemporal features, such as exploration ranges, path distributions, and stop regions, as supplementary information. Rich trajectory information supports typical mental map analyses of structural items and their interrelationships and in-depth inquiries, such as exploring the factors influencing the formation of structural information in mental maps. For example, Commins et al. [35] treated navigation strategies as variables in a navigation experiment, and distinguished between an allocentric strategy that relied on external cues and an egocentric strategy that depended on self-motion cues to collect trajectories. They further used path diagrams, heat maps, and quadrant diagrams to present the path distributions, stop regions, and dwell times within the trajectories (Fig. 2a). By analyzing these features, they demonstrated that the navigation strategies of individuals are important factors influencing the organization of landmarks and goal locations within mental maps. Similarly, Brunec et al. [36] further employed path distribution diagrams to present the movement densities and analyze the directional choices in trajectories (Fig. 2b). They identified the exploration strategies of individuals, including a systematic strategy characterized by broadly distributed movement densities and diverse directional choices and a restricted strategy characterized by locally concentrated movement densities and repetitive directional choices, revealing that different exploration strategies influenced the distribution of landmarks within mental maps. Researchers have further explored the formation mechanisms of structural items on mental maps. For example, Quesnot et al. [31] employed spatial distribution diagrams to present the trajectories combined with sketches (Fig. 2c), revealing that mental maps tend to form a hierarchical structure organized around the backbone of roads and landmarks by compressing redundant paths, simplifying overall shapes, and emphasizing the key landmarks and main roads.

Although drawn sketches and trajectories provide useful references for the analysis of structural information in mental maps, both types of data have inherent limitations. Drawn sketches are influenced by drawing ability, task instructions, symbol conventions, and the familiarity of individuals with the target environment, and distortions or omissions that may reflect limitations in external expressions rather than cognitive structures alone. Trajectories provide more objective behavioral records; however, movement patterns may also be affected by environmental constraints, route availability, navigation tasks, and temporary decisions. Therefore, visualizations based on sketches or trajectories should be interpreted carefully when inferring structural information from mental maps.

### Visualization for representing attribute information in mental maps

Visualizations that represent the attribute information of elements within mental maps aim to reveal subjective emotions (e.g., affection or fear), attitudes (e.g., support or opposition), or experiences (e.g., familiarity or unfamiliarity) of individuals regarding real-world objects. For example, a park presents a sense of familiarity in the mental map of an individual in urban neighborhoods, making the individual engage in relaxation and social interactions, whereas a campus has a sense of unfamiliarity, discouraging the individual from spending time in this location. Visualizing and analyzing these attributes helps scholars to explore why mental maps support daily human behaviors.

Two types of data, namely, textual descriptions and drawn sketches, are commonly adopted in existing studies to explore attribute information on mental maps. Textual descriptions are usually collected from verbal expressions or written texts of individuals, in which words and their collocations may reflect attribute information [44–47]. For example, one student described his mental map of campus as follows: “I am familiar with the north campus, but I have only been to the south campus once.” The sketches had the same data type as those described in Visualization for Depicting Structural Information in Mental Maps subsection. This subsection focuses on the annotations of drawn sketches that record different attribute information [40–43]. For example, circles were used to represent important landmarks, red was used to indicate familiar areas, and the word ‘affection’ was used to denote preferred buildings in an individual’s drawn sketch.

**Fig. 3:**
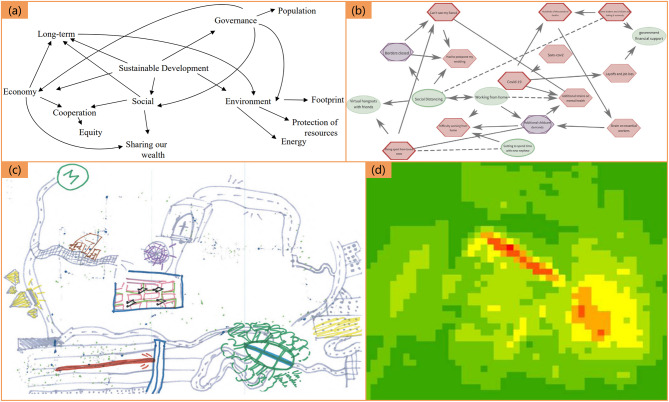
Representative examples of exploring attribute information in mental maps via visualizations. (**a**) [46] and (**b**) [44] are examples via textual descriptions; (**c**) [43] and (**d**) [42] are examples via drawn sketches

In existing studies, scholars have predominantly employed concept maps to visualize and analyze textual descriptions. Concept maps first extract words from textual descriptions and then construct a network model based on the extracted words, in which words are represented as nodes and the collocations or semantic relationships between words are represented as edges. Visualizing concept maps enables human viewers to identify important words and analyze the attributes contained in the words. For example, Lourdel et al. [46] employed a concept map to visualize the words and relationships extracted from an individual’s textual description in a discussion of sustainable development (Fig. 3a). The results revealed an individual’s attitudes toward sustainable development, for example, that the environment is the most important and the economy is marginalized. Reuter et al. [44] further conducted feature analysis to derive the attitudes of words within different textual descriptions. They subsequently used a colored concept map to represent words with attitudes (Fig. 3b), with green words indicating a positive attitude, red words indicating a negative attitude, and purple words indicating an attitude between positive and negative attitudes.

The annotations contained within drawn sketches help scholars to directly analyze attribute information in mental maps; as drawn sketches are an intuitive form of visual representation, they typically convey the attributes of elements within mental maps through colors, symbols, and texts. For example, Guelton [43] analyzed the annotated colors in a drawn sketch (Fig. 3c) and inferred that pink corresponds to familiar areas, whereas green and yellow correspond to preferred and unfavored areas, respectively. These interpretations were subsequently validated through interviews with the creators of the sketchs. Curtis et al. [42] further conducted a sentiment analysis of annotations in a drawn sketch, converted the analytical results into spatial raster data, and visualized them using a heatmap (Fig. 3d), in which the green, yellow, and red areas indicated neutral, mildly negative, and strongly negative emotions, respectively. By analyzing the heatmap and drawn sketches, they found that in the mental map of city gang communities, regions with frequent gang activities were characterized by negative emotions.

Textual descriptions and drawn sketches have been widely used to analyze attribute information in mental maps; however, both types of data have limitations. Textual descriptions can reveal the emotions, attitudes, and experiences of individuals; however, network-based concept maps may not fully preserve the richness of such subjective information as they commonly transform continuous and context-dependent expressions into discrete nodes and edges. Consequently, subtle emotional nuances and temporal changes may be lost during extraction and visualization. Drawn sketches provide more direct visual cues through colors, symbols, and texts; however, their annotations may be influenced by individual drawing habits, symbolic preferences, and the ambiguity of self-defined marks. These limitations indicate that the visualization of attribute information should be combined with contextual explanations or qualitative validation to avoid any oversimplification of the attributes contained within mental maps.

### Visualization for comparing mental maps

Visualizations to compare mental maps aim to reveal the differences and commonalities of structural and attribute information contained within multiple mental maps, which helps to explore the factors influencing the construction of mental maps. Existing studies predominantly focus on two dimensions: the comparison of mental maps between different individuals and between different groups. In this context, the mental map of a group is a collective representation formed by integrating the mental maps of multiple individuals within a group, thereby reflecting their common cognition.

**Fig. 4:**
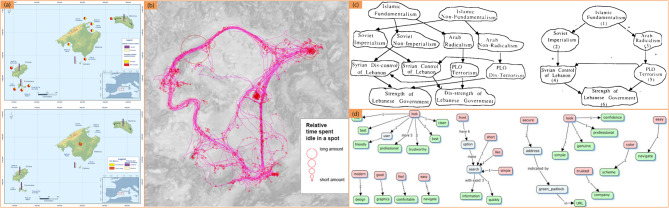
Representative examples of comparing mental maps via visualizations. (**a**) [48] and (**b**) [51] are examples comparing mental maps of individuals; (**c**) [56] and (**d**) [57] are examples comparing mental maps of groups

To analyze the differences and commonalities among multiple mental maps, scholars typically employ a visualization method to present mental maps individually, or to overlay mental maps within a single visual representation. For example, Binimelis et al. [48] designed a spatial visualization method (Fig. 4a) to present the drawn sketches of multiple individuals separately. They found that the shapes and relative positions of the islands in the drawn sketches were similar, whereas differences were observed in the numbers and distributions of cities and landmarks across different sketches. They conducted interviews with individuals, finding a positive correlation between the level of geographical knowledge of individuals and the number and spatial balance of cities and landmarks. This finding indicates that an individual’s knowledge affects the construction of mental maps. Ugwitz et al. [51] further visualized the trajectories of multiple individuals in a path diagram (Fig. 4b) and analyzed the stop regions and dwell times. They subsequently conducted interviews and cognitive style tests with individuals, revealing that individuals with a spatially oriented cognitive style had relatively low numbers of stop regions and short dwell times, while those with an object-oriented cognitive style had relatively high numbers of stop regions and long dwell times. This finding suggests that the cognitive style influences mental map construction.

Existing studies have predominantly used textual descriptions to construct the mental map of a group using a three-step process [54–57]. First, words were extracted from the textual descriptions of individuals within a group. Semantic and clustering analyses were conducted on the words. Finally, a network model was constructed to represent the shared cognition within the group. The visualization of multiple networks enables the intuitive comparison of mental maps between different groups. For example, Zhang et al. [56] constructed two networks of humanities and engineering groups for topic discussions; using concept maps to present the networks (Fig. 4c), they found that the humanities group had a relatively complex, word-rich network structure, whereas the engineering group had a relatively simple, word-limited network structure. This finding indicates that the disciplinary background influences the organizational structures of the elements within mental maps. Similarly, Albalawi et al. [57] constructed two networks of professional and nonprofessional groups in a safety-context discussion and employed concept maps to display the networks (Fig. 4d). They discovered that in the network of the professional group, safety threats and protective measures were regarded as core elements, while in the network of the nonprofessional group, visual and experiential features were considered the most important elements, with safety threats positioned on the periphery. This phenomenon indicates that professional knowledge influences the attributes of elements within different mental maps by shaping how individuals perceive security-versus usability-related elements.

Visual comparisons are useful for identifying differences and commonalities among mental maps; however, visual differences do not necessarily indicate cognitive differences. In individual-level comparisons, differences in drawn sketches or trajectories may be influenced by the drawing ability, task setting, environmental constraints, and data collection procedures. In group-level comparisons, aggregating individual descriptions can reveal shared cognition, but may also obscure minority perspectives and intra-group diversity. Moreover, overlaying or juxtaposing multiple mental maps can introduce visual clutter and render subtle structural or attribute differences difficult to observe.

## Mental maps for visualization

Mental maps are widely used as a theoretical foundation to guide visualization design, as their working mechanisms reflect the cognitive rules by which humans perceive and understand the external world. There are three primary mechanisms: element structuring [58–60], structural mapping [61, 62], and consistency maintenance [63–65]. These three mechanisms are summarized from behavioral, cognitive, and neuroscientific studies on mental maps, with their theoretical basis rooted in the organization of spatial and relational knowledge, mapping between external cues and internal representations, and continuous updating of mental representations during navigation and dynamic understanding. Each of these mechanisms can provide insights into human cognition to facilitate distinct visualization designs. Therefore, we classify the existing studies into three categories: (1) element structuring mechanism for visualization, (2) structural mapping mechanism for visualization, and (3) consistency maintenance mechanism for visualization. In practical visualization designs, the three mechanisms can sometimes work together, rather than appearing strictly in isolation. For example, dynamic visualization can map new data onto an existing cognitive structure, while maintaining visual consistency over time, thereby involving both structural mapping and consistency maintenance. In this survey, such studies were classified according to the primary design role of the mental map mechanism, namely, whether it predominantly guided element structuring, structural mapping, or consistency maintenance. Table 2 summarizes the references and representatives of these three categories, which are introduced in the following subsections.

**Table 2:** Relevant studies on mental maps for visualization

Category	Reference	Representative
Element structuring mechanism for visualization	[66–78]	[66, 69–71]
Structural mapping mechanism for visualization	[79–103]	[81, 86, 90, 93, 97]
Consistency maintenance mechanism for visualization	[104–128]	[116–121]

### Element structuring mechanism for visualization

The element structuring mechanism refers to the cognitive tendency of individuals to organize objects in the external world using preferred structures (e.g., hierarchical or networked structures), to construct mental maps [58–60]. This tendency primarily arises from common sense, and offers natural inspiration for visualization designs. For example, networked structures commonly emerge in human minds when perceiving friendships among persons, whereas hierarchical structures easily arise when recognizing management relationships among employees. As such, hierarchical visualization is a straightforward design choice for illustrating a company’s management mode.

**Fig. 5:**
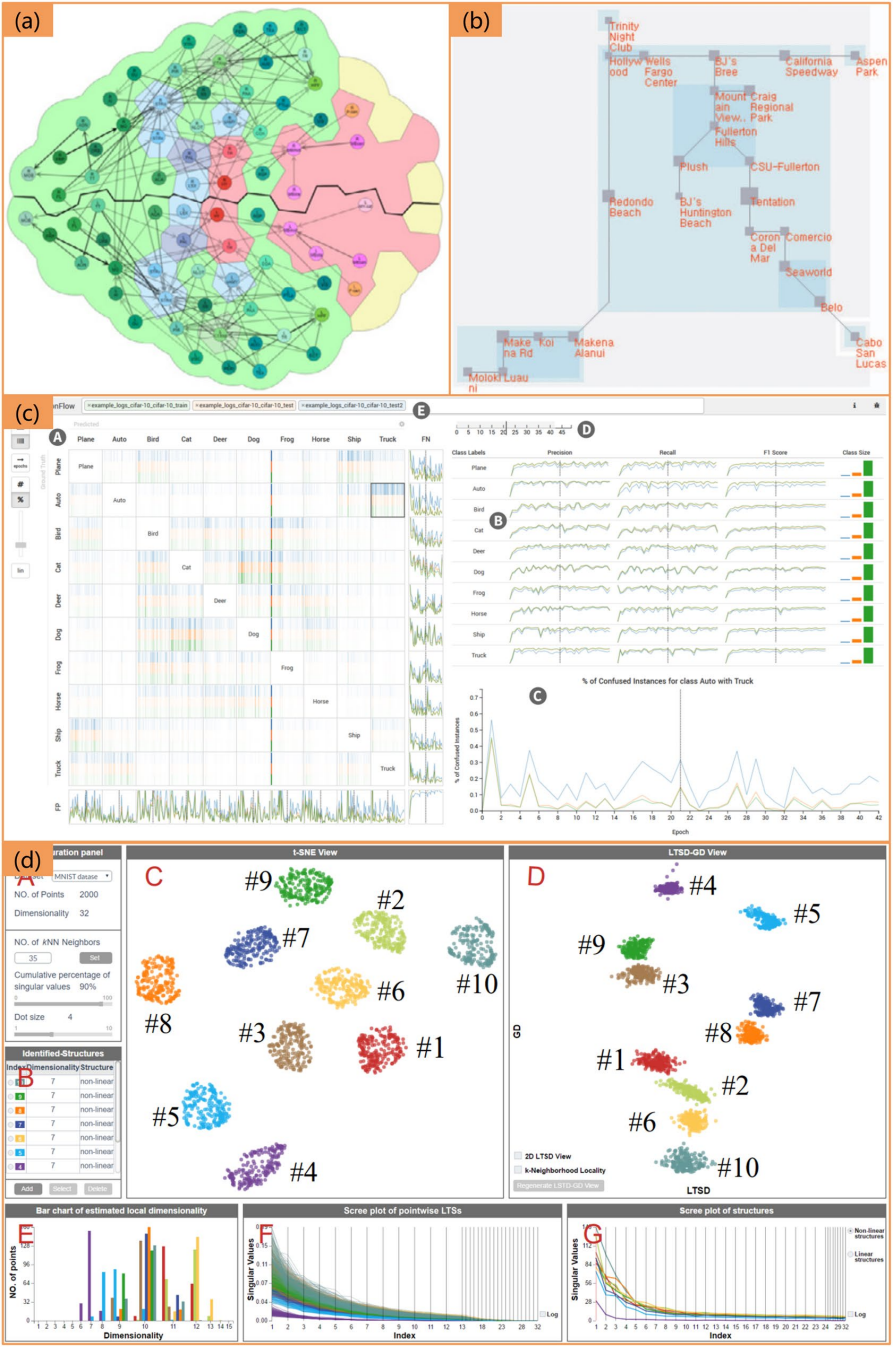
Representative examples using the element structuring mechanism to guide visualization designs, including a brain network visualization (**a**) [69], a geotagged data visualization (**b**) [66], a visual interface for model performance analysis (**c**) [70], and a visual interface for projection algorithm analysis (**d**) [71]

In existing studies, scholars have typically utilized the element structuring mechanism to design a specific arrangement method or layout algorithm to position different visual elements in a visualization. For example, Ganglberger et al. [69] visualized different brain networks. Considering that humans are inclined to understand anatomical structures according to spatial continuity, they designed a spatial-data-driven network layout instead of the commonly used force-directed network layout to preserve the spatial proximity relationships among different brain regions (Fig. 5a). Jo and Ryh [66] designed a visualization method to present personal geotagged data (Fig. 5b). They considered that humans tended to preserve relative positions, rather than precise locations when recalling tagged locations. Therefore, they adopted a metro-map-like design that arranged tagged locations in a relatively compact layout along several grid-like lines and used horizontal, vertical, or 45-degree connectors to indicate the visiting sequence between the locations.

Moreover, for a multi-view interface, each view can be regarded as a visual element, and the arrangement of multiple views in the interface may be treated as an element structure. Therefore, the element structuring mechanism can further provide a design perspective for interfaces containing multiple visualizations. As humans are inclined to compare different results of diverse running settings side by side, visual interfaces commonly adopt a juxtaposing structure to arrange multiple views and visualize the results to facilitate comparisons in many visual analysis studies aimed at algorithm or model tuning. For example, Hinterreiter et al. [70] designed a visual interface that juxtaposed multiple confusion matrix visualizations and multiple metric statistics visualizations to facilitate a comparison of the performances of different classification models on the same dataset (Fig. 5c). Similarly, Xia et al. [71] designed a visual interface (Fig. 5d) that juxtaposed different multiple-dimensional reduction projection visualizations to facilitate a comparison of the intra-cluster compactness and inter-cluster separability of clusters in the same dataset under different dimensional reduction projection algorithms.

The element structuring mechanism can make visual layouts more consistent with the familiar cognitive structures of users; however, excessive reliance on such structures may further constrain visualization design. When data relationships are interpreted as hierarchies, networks, or spatial proximities, aligning the layouts with these structures can improve the readability. However, for novel data types or exploratory analytical tasks, the existing mental map structures may be insufficient or even misleading. In such cases, forcing the data into familiar structures can limit the development of new visual metaphors, leading to suboptimal representations. Therefore, the element-structuring mechanism should be treated as a design heuristic, rather than a universal principle.

### Structural mapping mechanism for visualization

The structural mapping mechanism refers to the cognitive pattern by which individuals map different external world objects onto the structures of mental maps constructed in their minds [61, 62]. This cognitive pattern is widely used by experts in specialized domains to guide visualization design. Domain experts generally possess extensive knowledge and long-term practical experience, meaning they can form stable mental maps for analyzing and understanding domain-specific data. Therefore, when designing domain-oriented visualizations, it is crucial to engage in in-depth communication with domain experts to externalize their mental maps. The structures of externalized mental maps effectively guide the design of organizational structures for the visual elements within visualizations.

**Fig. 6:**
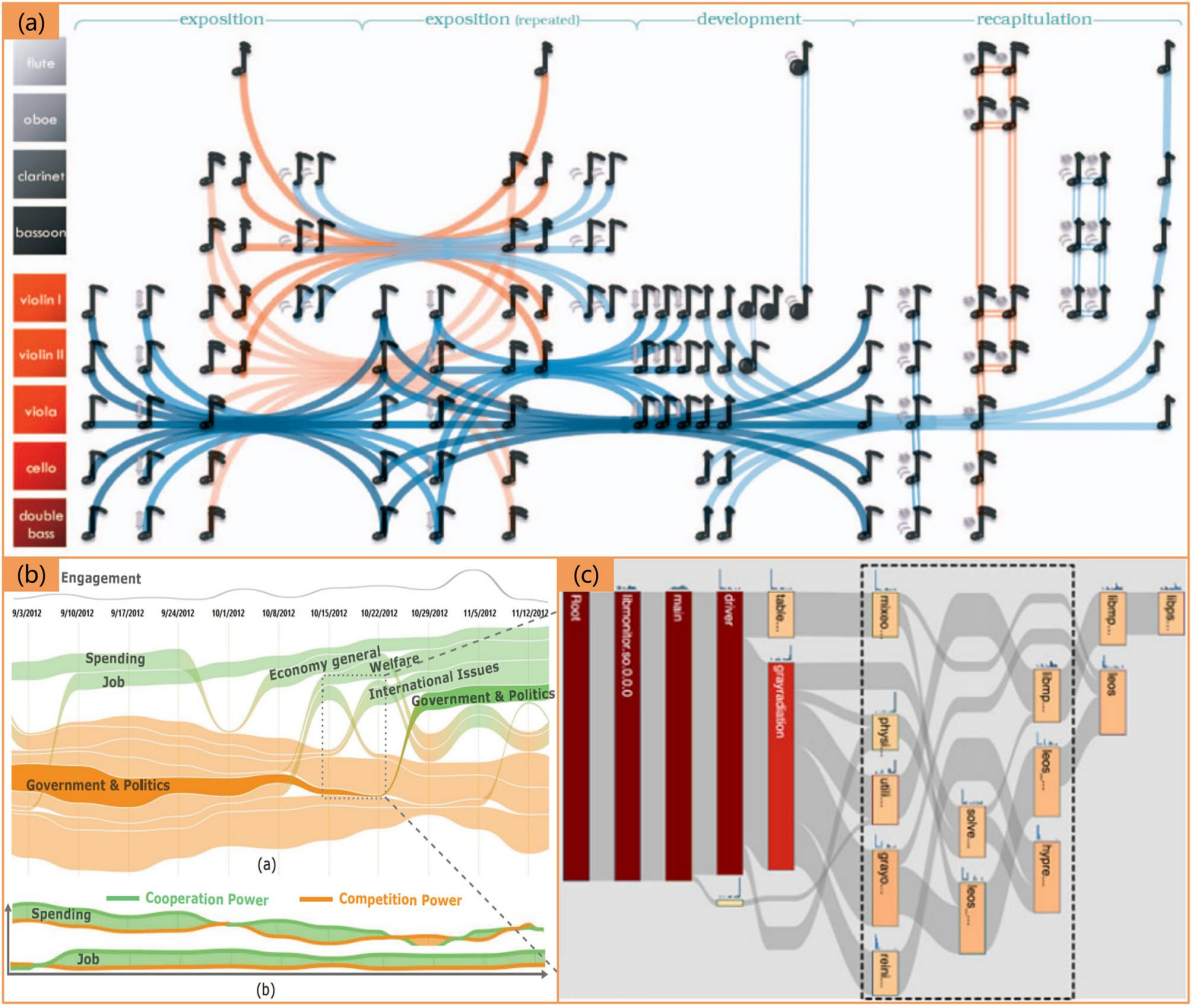
Representative examples of using the structural mapping mechanism to guide visualization designs in the music domain (**a**) [86], social media domain (**b**) [90], and performance analysis domain (**c**) [81]

Scholars have previously leveraged the structural mapping mechanism of mental maps to design numerous domain-oriented visualizations. For example, Chan et al. [86] designed a visualization method for classical polyphonic music based on the different mental maps of music experts. In interviews, they found that music experts typically possess a braided-structure mental map for polyphonic musical works in which multiple thematic cues carried by different voices are understood as musical semantic lines that continually intertwine, echo, and converge over time. Therefore, they adopted a braided structure in the visualization to organize the visual elements, explicitly presenting semantic relationships between themes and voices as interwoven lines (Fig. 6a). This approach highlights the structural associations among different thematic cues in polyphonic music, thereby enabling the structure of visual elements to align with the mental maps of experts, and strengthening their comprehension of complex musical semantics. Sun et al. [90] further designed a novel visualization method for topic analysis in the social media domain. Through interviews, they found that professional analysts generally have a flow-structured mental map for topic analysis, regarding topics and their evolutionary processes as dynamic streaming flows that continually merge and diverge over time. Based on this finding, they represented topics in the visualization as undulating fluid bands, and used threads to encode the influence trajectories of different types of opinions on these topics (Fig. 6b). This visualization design intuitively depicts topic evolution, and provides insights into the complex interactions of topics. Similarly, in the domain of program performance analysis, Nguyen et al. [81] found that performance analysis experts often have a hierarchical flow-structured mental map of program execution processes. These experts understand program execution as a structured flow that unfolds layer by layer along the calling chain, with performance metrics propagating across different functions. Accordingly, they designed a callflow visualization that arranges different function nodes vertically to preserve the call-tree hierarchy, and uses flow-like paths to represent the propagation of performance metrics along the calling chain (Fig. 6c). This visualization enables performance-analysis experts to simultaneously grasp both the calling hierarchy and the patterns of performance propagation, thereby helping them to efficiently identify performance bottlenecks and understand program behavior across different calling stages.

**Fig. 7:**
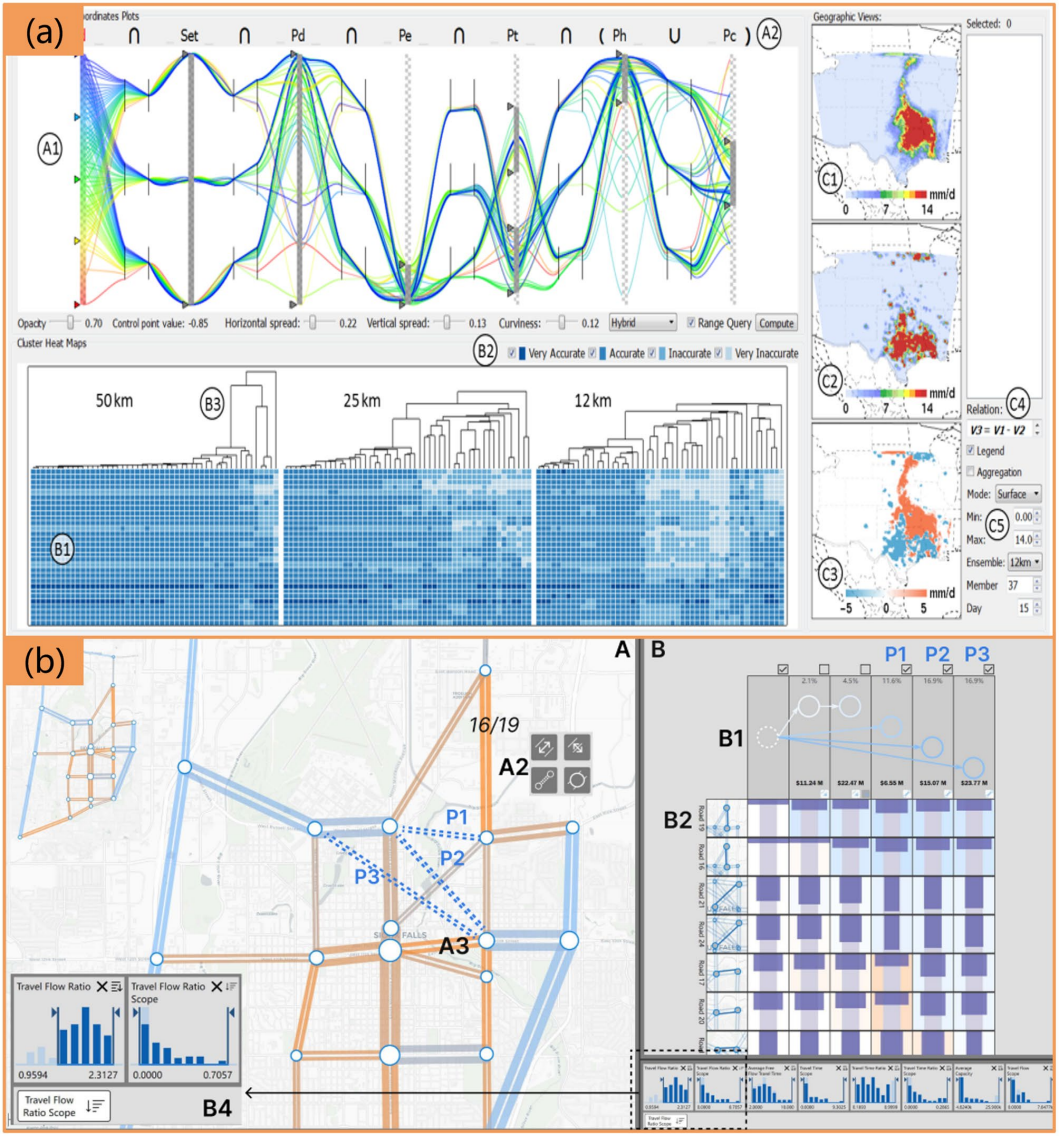
Representative examples of applying the structural mapping mechanism to guide visual interface designs in the climate domain (**a**) [93], and traffic domain (**b**) [97]

Some scholars have previously leveraged the structural mapping mechanism of mental maps to design different domain-oriented visual interfaces. For example, Wang et al. [93] designed a visual interface for climate parameter analysis based on the mental maps of climate experts (Fig. 7a), finding that climate experts typically possess a stage-structured mental map for climate parameter analysis. They first grasped the relationships among multiresolution parameters at a holistic level, then compared the similarities among simulation results under different parameter settings, and finally inspected the spatial distributions of specific regions in the simulation results. Consequently, they designed three visualization views in the interface corresponding to the three stages, where Views A, B, and C present the overall relational structure of the multiresolution parameters, different simulation results, and the spatial distributions of specific regions, respectively. These views align with the mental maps of climate experts, enabling them to analyze familiar pathways, thereby readily understanding the differences among simulation results and identifying important patterns. Similarly, Deng et al. [97] devised a visual interface for traffic planning based on the mental maps of traffic experts (Fig. 7b). Traffic experts generally adopt a two-stage mental map for traffic planning: first exploring candidate alternatives for alleviating congestion patterns and bottleneck road segments, and then comparing the performance metrics of multiple candidate alternatives to make decisions. Consequently, they designed two visualization views at the interface corresponding to the two stages. View A supported the experts in identifying existing traffic congestion, exploring feasible adjustment strategies, and interactively constructing candidate alternatives, while View B presented the performance of each candidate across multiple metrics, helping experts to compare and select candidate alternatives. These two views enabled experts to advance efficiently from alternative exploration to evaluation, while supporting iterative comparison and trade-off analysis across multiple candidate alternatives, ultimately enhancing the quality of traffic planning decisions.

The structural mapping mechanism depends heavily on the externalization of the mental maps of experts; however, these mental maps are not always stable, explicit, or shared by all experts. Rather, they are commonly shaped by professional experience, analytical habits, and domain conventions. Consequently, a visualization that closely follows the mental maps of one group of experts may fit familiar workflows, but may also overlook alternative analytical paths, different expert perspectives, or changing task requirements.

### Consistency maintenance mechanism for visualization

The consistency maintenance mechanism refers to the cognitive principle by which individuals understand dynamic objects in the external world by maintaining consistency in their mental maps [63–65]. For example, when observing time-varying vehicle flows, individuals often construct an initial mental map as a cognitive anchor based on the initially observed vehicles and their states. Individuals also tend to update the initial mental map for subsequently observed vehicles and their changed states instead of reconstructing a new mental map. This principle can greatly reduce the cognitive load associated with understanding dynamic objects, while also facilitating the rapid identification of changes. Consequently, this mechanism has been widely applied in the design of dynamic visualizations to present various time-varying data, such as statistical data [104–109], graph data [116–128], and hierarchical data [110–115]. Among existing studies, the visualization design of dynamic graph data has been discussed relatively extensively and in considerable depth. Therefore, this subsection uses dynamic graph visualizations as representatives to illustrate how the consistency maintenance mechanism guides the visualization designs.

Dynamic graphs are characterized by changes in the numbers of nodes and edges over time. Visualizing a dynamic graph in a node-link diagram requires the positions of nodes corresponding to unchanged data items across time slices to be fixed or nearly fixed to fit the consistency maintenance mechanism of the mental maps. In other words, the overall structure of the graph is perceived to be nearly unchanged over time, while the addition or deletion of nodes and edges can be easily observed. Because the generation of node-link diagrams relies heavily on graph layout algorithms, a consistency maintenance mechanism should be incorporated into the computation processes of different graph layout algorithms. Spring-electrical and stress models are widely used for designing graph-layout algorithms [129–138]. The two models have different optimization goals and computational processes, leading to distinct technical pathways for incorporating the consistency maintenance mechanism into dynamic graph visualizations.

**Fig. 8:**
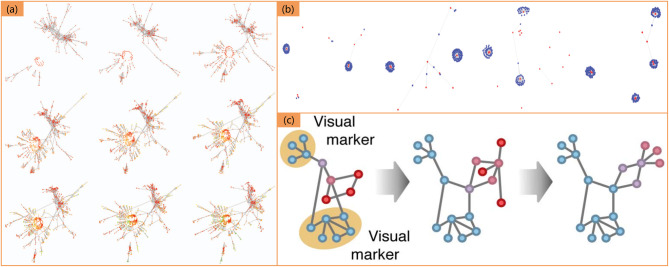
Representative examples of using the consistency maintenance mechanism to visualize different dynamic graph data via spring-electrical models, including a method incorporating a neighborhood-unchanged indicator (**a**) [116], a method with a node-importance indicator (**b**) [117], and a method introducing a node-age indicator (**c**) [118]

Spring-electrical models regard nodes as charged particles that produce repulsive forces repelling all nodes apart, and edges as springs that generate attractive forces that attract connected nodes together [129, 133–135]. The models iteratively move node positions by computing the resultant forces acting on the nodes when generating graph layouts, with the optimization goal of minimizing the resultant forces acting on all nodes. In dynamic graph visualizations using spring-electrical models, scholars typically design quantitative indicators to measure the mobility of nodes to achieve stability of the overall structure of a dynamic graph. For example, Frishman and Tal [116] proposed a dynamic graph layout method by introducing a neighborhood-unchanged indicator (Fig. 8a). This method first identified the changes in the neighborhoods of each node between the previous and current time slices of a dynamic graph and then assigned a neighborhood-unchanged value to each node. For nodes with high indicator values, the influence of the resultant forces was significantly reduced to limit their movement during the layout process. Zhou et al. [117] further designed a node importance indicator for dynamic graph visualization (Fig. 8b). This indicator reflected the visual importance of a node by computing its centrality. The resultant forces acting on important nodes are proportionally reduced to constrain node movements. Gorochowski et al. [118] further proposed a node-age indicator that defines the age of a node using the number of time slices in which the node occurs (Fig. 8c). The resultant forces acting on the older nodes were largely attenuated to avoid large positional changes in these nodes.

**Fig. 9:**
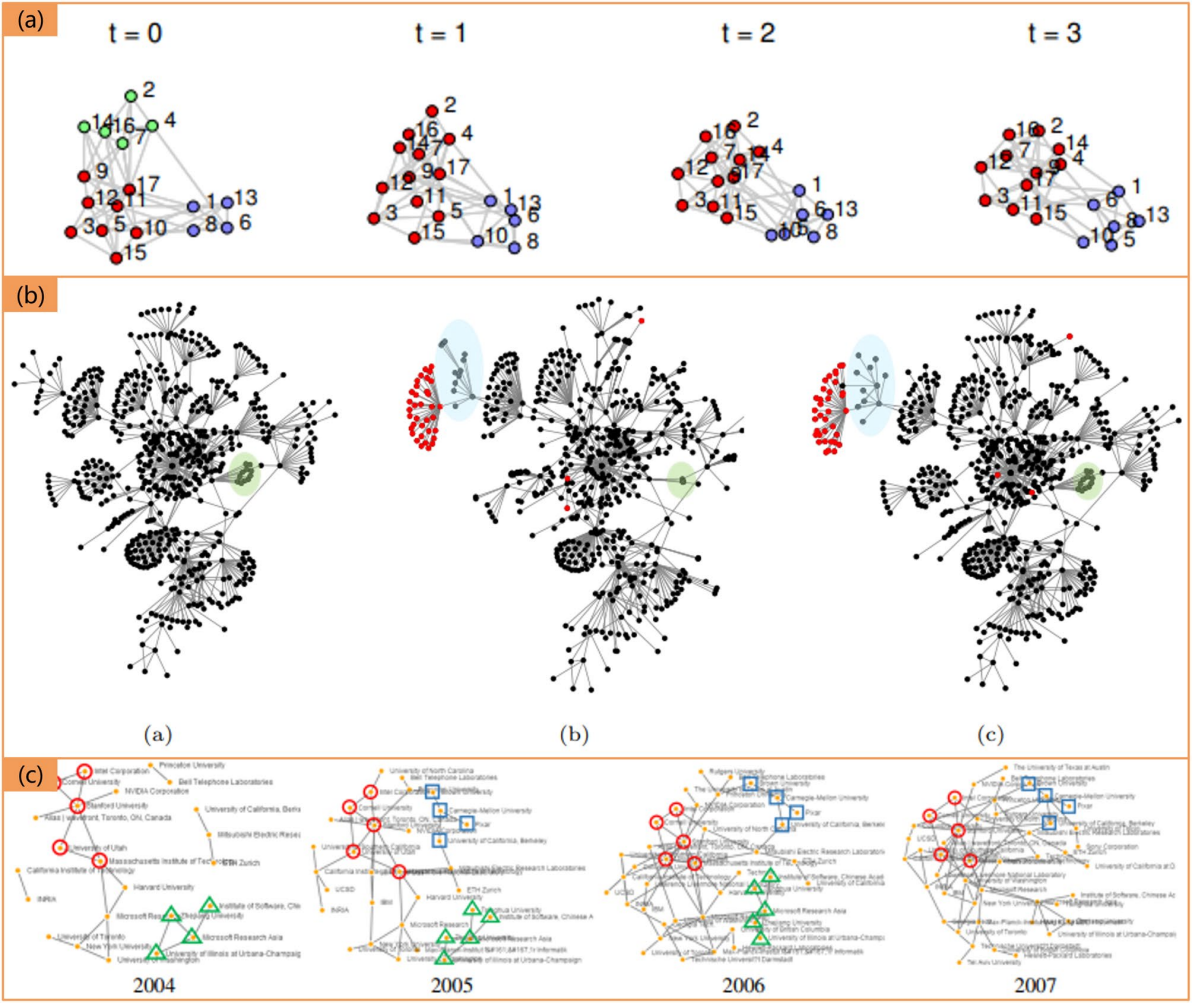
Representative examples of using the consistency maintenance mechanism to visualize dynamic graph data via stress models, including a method introducing a temporal constraint (**a**) [119], a method with a stability constraint (**b**) [120], and a method incorporating an edge-length constraint (**c**) [121]

Stress models construct an energy function with a predefined layout-distance constraint to generate a graph layout. Graph-theoretical distance is a commonly used expected layout distance between two nodes. A large deviation between actual and expected layout distances corresponds to high energy [130, 132, 135]. The models iteratively change the node positions to minimize energy when computing an expected graph layout. In dynamic graph visualizations via stress models, scholars typically add additional constraints to the energy function to control the changes in node positions. For example, Xu et al. [119] proposed a stress-model-based dynamic graph layout method by introducing a temporal constraint (Fig. 9a) which restricts the positional deviations of the nodes that exist in two adjacent time slices. The constraint and a penalty term are added to the energy function to characterize the cost of node position changes between adjacent time slices. If a node undergoes a large displacement, the penalty term increases the layout energy to suppress the large node displacements during the layout process. Sheng et al. [120] further designed a stability constraint utilizing the inverse Markov process to evaluate the extent to which changes in node positions affect the overall graph layout (Fig. 9b). The stability constraint was incorporated into the energy function as a penalty term. When node position changes with high stability values, high penalties prevent these changes during the layout process. Che et al. [121] proposed a dynamic graph layout method that incorporates an edge-length constraint (Fig. 9c). This method considers the edge lengths of the previous time slice as a constraint for generating the layout of the current time slice. The constraint is subsequently added to the energy function as a penalty term to penalize large changes in edge lengths, thereby preventing any large positional changes between edge-connected nodes.

The consistency maintenance mechanism provides useful guidance for dynamic visualizations, but strong consistency does not always lead to better visual representations. In dynamic graph layouts, preserving node positions across time can help users to track graph evolution; however, excessive constraints on node movement can reduce the readability of individual time slices or obscure newly formed structures. Therefore, the use of this mechanism involves a tradeoff between temporal stability and layout readability, and the appropriate balance depends on the analytical task and the characteristics of the graph changes.

## Research opportunities and challenges

In the previous subsections, we systematically summarized the results of existing studies in the intersection of mental maps and visualization from two perspectives, namely, visualization for mental maps and mental maps for visualization. These studies demonstrated substantial potential for the development of this interdisciplinary field. The visualization tools discussed in Visualization for Mental Maps section, such as those based on drawn sketches, trajectories, and textual descriptions, can make implicit cognitive processes more observable, including how individuals or groups organize elements, associate attributes with external objects, and update mental maps for distinct tasks. Conversely, the element structuring, structural mapping, and consistency maintenance mechanisms discussed in Mental Maps for Visualization section can provide cognitive perspectives for the interpretation and refinement of these visualization tools, such as selecting appropriate visual encoding, designing layouts, and supporting comparisons across mental maps. In this subsection, we discuss the future research opportunities and challenges at the intersection of mental maps and visualization to provide insights for relevant researchers and practitioners.

**Evaluation criteria for visualizations of mental maps.** Although existing visualizations of mental maps take diverse forms, clear criteria for evaluating the extent to which these visualizations comprehensively represent mental maps are currently lacking. This makes it difficult to compare different visualization methods, or to determine whether visualization effectively supports the analysis of mental maps. Future research could explore unified evaluation criteria for the visualization of mental maps, enabling a more systematic assessment of their effectiveness.

To account for individual differences in mental maps, unified evaluation criteria could be designed as a flexible framework, rather than as a fixed set of indicators. One feasible direction would be to define several general evaluation dimensions, while allowing specific indicators to be adjusted according to users, tasks, and contexts. For example, general dimensions may include the completeness of represented information, interpretability of visual encodings, consistency between visual representations and user cognition, and the usefulness of visualizations for analysis tasks. Under these dimensions, individual differences, such as spatial ability, familiarity with the environment, cultural background, and domain knowledge, can be considered contextual factors rather than treated as noise. Thus, the evaluation criteria can provide a common basis for comparing visualization methods while preserving the diversity of the mental maps.

Advancements in this field still face several challenges. Specifically, visual encoding and layout strategies vary across visualizations, making it difficult to apply the same criteria across all cases. Moreover, mental maps are often subjective and context-dependent, meaning that the evaluation results may differ across individuals, even when the same visualization is applied. Therefore, future evaluation criteria should balance general applicability with sensitivity to individual differences rather than assuming that a single fixed standard can fully capture all cognitive characteristics of mental maps.

**Standardized applications of mental map mechanisms.** Although mental map mechanisms have been widely applied to guide visualization designs, most applications remain at the level of empirical summaries and case-driven explorations, thereby lacking unified and transferable guidance strategies for visualization designs. Future research should therefore investigate the applicability conditions and processes of mental mapping mechanisms to form standardized guidance strategies for visualization design.

One possible direction would be to establish a general mechanism-to-design framework. Such a framework could clarify how mental map mechanisms correspond to different visualization requirements, such as data organization, visual mapping, layout arrangement, interaction design, and temporal continuity. Instead of treating mental map mechanisms only as an abstract design inspiration, future research could further translate them into reusable design principles or patterns. This would make it easier to compare how the same mechanism works across different domains, and how different mechanisms can be combined in complex visualization systems.

Nevertheless, this direction is confronted with two critical challenges. First, mental map mechanisms inherently exhibit a certain degree of semantic flexibility, making it difficult to define their functional scope precisely. Second, the targets of visualization designs vary across scenarios, making it difficult to construct processes that are suitable across scenarios. Therefore, standardized applications should not be understood as fixed design rules. Instead, they should provide adaptable guidance to aid researchers in judging when a mechanism is applicable, how it can be translated into a visualization design, and what limitations may arise in specific scenarios.

**Mental maps for automatic visualization generations.** Existing studies have adopted mental maps as a theoretical foundation to guide visualization designs, aiming to enhance the alignment between visualizations and human cognition. In recent years, following the rapid development of automatic visualization generation techniques [139–141], mental maps have the potential to serve as a cognitive-level theoretical foundation for such generation processes. However, incorporating mental maps into automatic visualization generation currently remains a critical challenge. Mental maps are abstract internal representations, and converting them into semantic or feature representations required by relevant models still warrants in-depth investigation.

To incorporate mental maps into automatic visualization generations, one central task that must be accomplished is to bridge the gap between internal cognitive representations and computational design representations. First, mental maps need to be externalized from available data, such as drawn sketches, trajectories, textual descriptions, interaction records, or interviews. The externalized information can then be transformed into structured representations such as elements, relations, attributes, task intentions, user preferences, or cognitive constraints. These structured representations can be further incorporated into automatic visualization generation models to guide the selection of visual encoding, layout strategies, and interaction designs. Thus, mental maps can move from abstract cognitive concepts to operational inputs to generate visualizations.

Large language models (LLMs) may provide useful support for addressing this challenge. They can help extract cognitive intentions, semantic relations, and design requirements from textual descriptions or user feedback, and further assist in generating candidate visualization designs based on these structured representations. The use of LLMs will also require careful validation, including assessment of whether the extracted mental map representations are reliable, whether biased or incomplete cognitive structures are reinforced, and whether the generated visualizations truly improve the cognitive alignment and task performance.

## Conclusions

In the present study, we presented a survey of the intersection of mental maps and visualization. We systematically reviewed existing studies in this interdisciplinary field and classified them into two categories: visualization for mental maps and mental maps for visualization. In the category of visualization for mental maps, we comprehensively introduced the principal methods for transforming mental maps into visual representations, along with their significance. In the category of mental maps for visualization, we explicitly elaborate how mental maps serve as a theoretical foundation from a human perspective to guide visualization designs. Based on an analysis of existing studies, we discuss future research opportunities and challenges in this interdisciplinary field. We hope that this survey will provide insights into the intersection of mental maps and visualization.

## Data Availability

Not applicable.

## Abbreviations

HCI: Human-computer interaction
LLM: Large language model

## References

[1] Tolman EC (1948) Cognitive maps in rats and men. Psychological Rev 55(4):189–208. 10.1037/h0061626 18870876 10.1037/h0061626

[2] Lynch K (1964) The Image of the City. MIT Press, Cambridge

[3] Stea D (2017) Image and environment: cognitive mapping and spatial behavior. Routledge, New York

[4] Kitchin RM (1994) Cognitive maps: what are they and why study them? J Environ Psychol 14(1):1–19. 10.1016/S0272-4944(05)80194-X

[5] Tversky B (1993) Cognitive maps, cognitive collages, and spatial mental models. In: Proceedings of the international conference on spatial information theory: a theoretical basis for GIS, Springer, Elba Island, 19–22 September 1993. 10.1007/3-540-57207-4_2

[6] Montello DR (2002) Cognitive map-design research in the twentieth century: theoretical and empirical approaches. Cartogr Geogr Inf Sci 29(3):283–304. 10.1559/152304002782008503

[7] McCormick BH (1988) Visualization in scientific computing. ACM SIGBIO Newslett 10(1):15–21. 10.1145/43965.43966

[8] Foley JD, van Dam A, Feiner SK, Hughes JF (1996) Computer graphics: principles and practice, 2nd edn. Addison-Wesley, Boston

[9] Shneiderman S (1983) Direct manipulation: a step beyond programming languages. Computer 16(8):57–69. 10.1109/MC.1983.1654471

[10] Robertson GG, Mackinlay JD, Card SK (1991) Cone trees: animated 3D visualizations of hierarchical information. In: Proceedings of the SIGCHI conference on human factors in computing systems, ACM, New Orleans, 27 April–2 May. 10.1145/108844.108883

[11] Shneiderman B (2003) The eyes have it: A task by data type taxonomy for information visualizations. In: Bederson BB, Shneiderman B (eds) The craft of information visualization. Elsevier, Amsterdam, pp 364–371. 10.1016/B978-155860915-0/50046-9

[12] Nielson GM, Hagen H, Müller H (1997) Scientific visualization: overviews, methodologies, techniques. Institute of Visual Computing & Human-Centered Technology, Vienna

[13] Gershon N, Eick SG, Card S (1998) Information visualization. Interactions 5(2):9–15. 10.1145/274430.274432

[14] Card SK, Mackinlay JD, Shneiderman B (1999) Readings in information visualization: using vision to think. Morgan Kaufmann, San Francisco

[15] Munzner T (2025) Visualization analysis and design. In: Proceedings of the special interest group on computer graphics and interactive techniques conference courses, ACM, Vancouver, 10–14 August 2025. 10.1145/3721241.3733989

[16] Scaife M, Rogers Y (1996) External cognition: how do graphical representations work? International Journal of Human-Computer Studies 45(2):185–213. 10.1006/ijhc.1996.0048

[17] Newell WH (2001) A theory of interdisciplinary studies. Issues Integr Stud 19:1–25

[18] Newell WH (2013) The state of the field: interdisciplinary theory. Issues Interdiscip Stud 31:22–43

[19] Klein JT, Newell WH (1996) Advancing interdisciplinary studies. In: Gaff JG, Ratcliff JL, Associates (eds) Handbook of the undergraduate curriculum. A comprehensive guide to purposes, structures, practices, and change, Jossey-Bass, San Francisco, pp 393–415

[20] Newell WH, Green WJ (1982) Defining and teaching interdisciplinary studies. Improving College and University Teaching 30(1):23–30. 10.1080/00193089.1982.10533747

[21] Freundschuh SM, Egenhofer MJ (1997) Human conceptions of spaces: implications for GIS. Transactions In GIS 2(4):361–375. 10.1111/j.1467-9671.1997.tb00063.x

[22] Hegarty M, Waller DA (2005) Individual differences in spatial abilities. In: Shah P, Miyake A (eds) The cambridge handbook of visuospatial thinking. Cambridge University Press, Cambridge, pp 121–169. 10.1017/CBO9780511610448.005

[23] Stryjewsja D, Kwoka K, Szymanowska P, Janda-Debek B (2016) Cogmap analyst–a quantitative analysis of the structure and content characteristics of sketch drawings of cognitive maps of urbanized spaces. Pol J Appl Psychol 14(4):9–34. 10.1515/pjap-2015-0065

[24] Kim J, Vasardani M, Winter S (2016) From descriptions to depictions: a dynamic sketch map drawing strategy. Spat Cognit Comput 16(1):29–53. 10.1080/13875868.2015.1084509

[25] Askarizad R, He JL (2022) Perception of spatial legibility and its association with human mobility patterns: an empirical assessment of the historical districts in Rasht, Iran. Int J Environ Res Public Health 19(22):15258. 10.3390/ijerph192215258 10.3390/ijerph192215258 PMC9690665 36429976

[26] Niman EM, Wejang HEA (2023) Students’ spatial thinking toward the school environment in Indonesia. Interdiscip J Educ Res 5:61–71. 10.38140/ijer-2023.vol5.06

[27] Chumakov R, Ryabinin KV, Belousov KI (2022) Visualization of mental map representation patterns. In: Proceedings of the 32nd international conference on computer graphics and vision, Ryazan State Radio Engineering University, Ryazan, 19–22 September 2022. 10.20948/graphicon-2022-248-274

[28] Dickmann F (2012) City maps versus map-based navigation systems – an empirical approach to building mental representations. Cartogr J 49(1):62–69. 10.1179/1743277411Y.0000000018

[29] Hátlová K, Hanus M (2020) A systematic review into factors influencing sketch map quality. ISPRS Int Jo Geo-Inf 9(4):271. 10.3390/ijgi9040271

[30] Tu Huynh N, Doherty ST (2007) Digital sketch-map drawing as an instrument to collect data about spatial cognition. Cartographica 42(4):285–296. 10.3138/carto.42.4.285

[31] Quesnot T, Guelton B (2025) Remembering routes: confronting spatial behaviours and sketch maps in individual and collective contexts. Front Psychol 16:1541363. 10.3389/fpsyg.2025.1541363 10.3389/fpsyg.2025.1541363 PMC12082656 40386670

[32] Coates P, Derix C, Lau T, Parvin T, Puusepp R (2005) Topological approximations for spatial representations. In: Proceedings of the 8th generative art conference, Generative Design Lab Milan Polytechnic University, Milan, 1 January 2005

[33] Kessler F, Frankenstein J, Rothkopf CA (2024) Human navigation strategies and their errors result from dynamic interactions of spatial uncertainties. Nat Commun 15(1):5677. 10.1038/s41467-024-49722-y 38971789 10.1038/s41467-024-49722-y PMC11227593

[34] König SU, Clay V, Nolte D, Duesberg L, Kuske N, König P (2019) Learning of spatial properties of a large-scale virtual city with an interactive map. Front Hum Neurosci 13:240. 10.3389/fnhum.2019.00240 31354457 10.3389/fnhum.2019.00240 PMC6636411

[35] Commins S, Duffin J, Chaves K, Leahy D, Corcoran K, Caffrey M et al (2020) NavWell: a simplified virtual-reality platform for spatial navigation and memory experiments. Behav Res 52(3):1189–1207. 10.3758/s13428-019-01310-5 10.3758/s13428-019-01310-5 31637666

[36] Brunec IK, Nantais MM, Sutton JE, Epstein RA, Newcombe NS (2023) Exploration patterns shape cognitive map learning. Cognition 233:105360. 10.1016/j.cognition.2022.105360 10.1016/j.cognition.2022.105360 PMC9983142 36549130

[37] Bock O (2025) An experimental study on the formation of spatial cognitive maps in humans. Appl Sci 15(13):7234. 10.3390/app15137234

[38] Ottink L, Hoogendonk M, Doeller CF, van der Geest TM, Van Wezel RJA (2021) Cognitive map formation through haptic and visual exploration of tactile city-like maps. Sci Rep 11(1):15254. 10.1038/s41598-021-94778-1 10.1038/s41598-021-94778-1 PMC8316501 34315940

[39] Cen DL, Teichert E, Hodgetts CJ, Gruber MJ (2024) Curiosity shapes spatial exploration and cognitive map formation in humans. Commun Psychol 2(1):129. 10.1038/s44271-024-00174-6 39738810 10.1038/s44271-024-00174-6 PMC11685098

[40] Ciobanu C (2008) The mental map of neighborhoods in Bucharest introductive study of mental geography. J Stud Res Hum Geogr 2(1):25–34

[41] Chumakov RV, Ryabinin KV, Belousov KI, Duan J (2021) Creative map studio: a platform for visual analytics of mental maps. Sci Visualization 13(2):79–93. 10.26583/sv.13.2.06

[42] Curtis JW, Shiau E, Lowery B, Sloane D, Hennigan K, Curtis A (2014) The prospects and problems of integrating sketch maps with geographic information systems to understand environmental perception: a case study of mapping youth fear in Los Angeles gang neighborhoods. Environ Plann B Plann Des 41(2):251–271. 10.1068/b38151

[43] Guelton B (2023) “Mental maps”: between memorial transcription and symbolic projection. Front Psychol 14:1142238. 10.3389/fpsyg.2023.1142238 10.3389/fpsyg.2023.1142238 PMC10086158 37057159

[44] Reuter L, Mansell J, Rhea C, Kiesel A (2022) Direct assessment of individual connotation and experience: an introduction to cognitive-affective mapping. Polit Life Sci 41(1):131–139. 10.1017/pls.2021.31 10.1017/pls.2021.31 36877115

[45] Barreto IF, Crescitelli E, Figueiredo JCB (2015) Relationship marketing results: proposition of a cognitive mapping model. Rev Bras Gest Neg 17(58):1371–1389. 10.7819/rbgn.v17i58.2692

[46] Lourdel N, Gondran N, Laforest V, Debray B, Brodhag C (2007) Sustainable development cognitive map: a new method of evaluating student understanding. Int J Sustain Higher Educ 8(2):170–182. 10.1108/14676370710726634

[47] Novak JD, Cañas AJ (2008) The theory underlying concept maps and how to construct and use them. Technical report IHMC CmapTools 2006–01 Rev 2008-01, Florida Institute for Human and Machine Cognition

[48] Binimelis Sebastián J, Pla-Sanchís C, Serrano-Varón J, Sánchez Casado M (2023) The use of mental maps in the assessment of geographic knowledge: form and content of map sketches drawn by last year primary education students in the Balearic islands (Spain). Geogr Pol 96(2):279–297. 10.7163/GPol.0256

[49] Kröse BJA, Booij O, Zivkovic Z (2007) A geometrically constrained image similarity measure for visual mapping, localization and navigation. In: Proceedings of the 3rd European conference on mobile robots, EMCR, Freiburg, 19–21 September 2007

[50] Cheng BJ, Wunderlich A, Gramann K, Lin ER, Fabrikant SI (2022) The effect of landmark visualization in mobile maps on brain activity during navigation: a virtual reality study. Front Virtual Real 3:981625. 10.3389/frvir.2022.981625

[51] Ugwitz P, Juřík V, Herman L, Stachoň Z, Kubíček P, Šašinka Č (2019) Spatial analysis of navigation in virtual geographic environments. Appl Sci 9(9):1873. 10.3390/app9091873

[52] Meneghetti C, Pazzaglia F (2021) Navigating in virtual environments: does a map or a map-based description presented beforehand help? Brain Sci 11(6):773. 10.3390/brainsci11060773 34200894 10.3390/brainsci11060773 PMC8230476

[53] Fabroyir H, Teng WC (2018) Navigation in virtual environments using head-mounted displays: allocentric vs. egocentric behaviors. Comput Hum Behav 80:331–343. 10.1016/j.chb.2017.11.033

[54] Afonso-Jaco A, Adam E, Katz BFG (2024) On prior visual experience in mental map navigation using allocentric and egocentric perspectives in the visually impaired. Quart J Exp Psychol, 17470218241286704. 10.1177/17470218241286704 10.1177/17470218241286704 39294108

[55] Fontaine S, Edwards G, Tversky B, Denis M (2005) Expert and non-expert knowledge of loosely structured environments. In: Proceedings of the 7th international conference on spatial information theory, Springer, Ellicottville, 14–18 September 2005. 10.1007/11556114_23

[56] Zhang WR, Chen SS, Bezdek JC (1989) Pool2: a generic system for cognitive map development and decision analysis. IEEE Trans Syst Man Cybern 19(1):31–39. 10.1109/21.24529

[57] Albalawi T, Ghazinour K, Melton A (2017) Security mental model: cognitive map approach. In: Proceedings of the international conference on computational science and computational intelligence (CSCI), IEEE, Las Vegas,14–16 December 2017. 10.1109/CSCI.2017.12

[58] Epstein RA, Patai EZ, Julian JB, Spiers HJ (2017) The cognitive map in humans: spatial navigation and beyond. Nat Neurosci 20(11):1504–1513. 10.1038/nn.4656 29073650 10.1038/nn.4656 PMC6028313

[59] Behrens TEJ, Muller TH, Whittington JCR, Mark S, Baram AB, Stachenfeld KL et al (2018) What is a cognitive map? Organizing knowledge for flexible behavior. Neuron 100(2):490–509. 10.1016/j.neuron.2018.10.002 30359611 10.1016/j.neuron.2018.10.002

[60] Ramadier T, Moser G (1998) Social legibility, the cognitive map and urban behaviour. J Environ Psychol 18(3):307–319. 10.1006/jevp.1998.0099

[61] Moser EI, Moser MB, McNaughton BL (2017) Spatial representation in the hippocampal formation: a history. Nat Neurosci 20(11):1448–1464. 10.1038/nn.4653 29073644 10.1038/nn.4653

[62] Watabe T, Niitsuma M (2013) Mental map generation assistance tool using relative pitch difference and angular information for visually impaired people. In: Proceedings of the IEEE 4th international conference on cognitive infocommunications (CogInfoCom), IEEE, Budapest, 2–5 December 2013. 10.1109/CogInfoCom.2013.6719252

[63] McNaughton BL, Battaglia FP, Jensen O, Moser EI, Moser MB (2006) Path integration and the neural basis of the ‘cognitive map’. Nat Rev Neurosci 7(8):663–678. 10.1038/nrn1932 16858394 10.1038/nrn1932

[64] Moser EI, Kropff E, Moser MB (2008) Place cells, grid cells, and the Brain’s spatial representation system. Annu Rev Neurosci 31(1):69–89. 10.1146/annurev.neuro.31.061307.090723 18284371 10.1146/annurev.neuro.31.061307.090723

[65] Krupic J, Bauza M, Burton S, O’Keefe J (2018) Local transformations of the hippocampal cognitive map. Science 359(6380):1143–1146. 10.1126/science.aao4960 29590044 10.1126/science.aao4960 PMC6331044

[66] Jo H, Ryu JH (2010) Placegram: a diagrammatic map for personal geotagged data browsing. IEEE Trans Visual Comput Graphics 16(2):221–234. 10.1109/TVCG.2009.68 10.1109/TVCG.2009.68 20075483

[67] Borkin MA, Yeh CS, Boyd M, Macko P, Gajos KZ, Seltzer M et al (2013) Evaluation of filesystem provenance visualization tools. IEEE Trans Visual Comput Graphics 19(12):2476–2485. 10.1109/TVCG.2013.155 10.1109/TVCG.2013.155 24051814

[68] Ten Caat M, Maurits NM, Roerdink Jbtm (2007) Design and evaluation of tiled parallel coordinate visualization of multichannel EEG data. IEEE Trans Visual Comput Graphics 13(1):70–79. 10.1109/TVCG.2007.9 10.1109/TVCG.2007.9 17093337

[69] Ganglberger F, Wißmann M, Wu HY, Swoboda N, Thum A, Haubensak W et al (2022) Spatial-data-driven layouting for brain network visualization. Computers Graphics 105:12–24. 10.1016/j.cag.2022.04.014

[70] Hinterreiter A, Ruch P, Stitz H, Ennemoser M, Bernard J, Strobelt H et al (2022) Confusionflow: a model-agnostic visualization for temporal analysis of classifier confusion. IEEE Trans Visual Comput Graphics 28(2):1222–1236. 10.1109/TVCG.2020.3012063 10.1109/TVCG.2020.3012063 32746284

[71] Xia JZ, Ye FJ, Chen W, Wang YS, Chen WF, Ma YX et al (2018) Ldsscanner: exploratory analysis of low-dimensional structures in high-dimensional datasets. IEEE Trans Visual Comput Graphics 24(1):236–245. 10.1109/TVCG.2017.2744098 10.1109/TVCG.2017.2744098 28866522

[72] Arleo A, Didimo W, Liotta G, Miksch S, Montecchiani F (2022) Influence maximization with visual analytics. IEEE Trans Visual Comput Graphics 28(10):3428–3440. 10.1109/TVCG.2022.3190623 10.1109/TVCG.2022.3190623 35830402

[73] Wang JP, Zhang W, Yang H, Yeh CCM, Wang L (2022) Visual analytics for RNN-based deep reinforcement learning. IEEE Trans Visual Comput Graphics 28(12):4141–4155. 10.1109/TVCG.2021.3076749 10.1109/TVCG.2021.3076749 33929961

[74] Shi L, Hu JN, Tan ZH, Tao J, Ding JY, Jin Y et al (2022) Mvnet: multi-variate multi-view brain network comparison over uncertain data. IEEE Trans Visual Comput Graphics 28(12):4640–4657. 10.1109/TVCG.2021.3098123 10.1109/TVCG.2021.3098123 34283716

[75] Zhang JZ, Lv HJ, Niu ZB (2025) AnchorTextVis: a visual analytics approach for fast comparison of text embeddings. IEEE Comput Grap Appl 45(6):29–43. 10.1109/MCG.2025.3598262 10.1109/MCG.2025.3598262 40802625

[76] Chen X, Zeng W, Lin YN, Ai-Maneea HM, Roberts J, Chang R (2021) Composition and configuration patterns in multiple-view visualizations. IEEE Trans Visual Comput Graphics 27(2):1514–1524. 10.1109/TVCG.2020.3030338 10.1109/TVCG.2020.3030338 33048683

[77] Xiong WL, Yu CJ, Shi C, Zheng YX, Wang XP, Hu YP et al(2024) V4RIN: visual analysis of regional industry network with domain knowledge. Vis Comput Ind Biomed Art 7(1):11. 10.1186/s42492-024-00164-9 38748079 10.1186/s42492-024-00164-9 PMC11096142

[78] Garcia R, Munz T, Weiskopf D (2021) Visual analytics tool for the interpretation of hidden states in recurrent neural networks. Vis Comput Ind Biomed Art 4(1):24. 10.1186/s42492-021-00090-0 34585277 10.1186/s42492-021-00090-0 PMC8479019

[79] Xin R, Ai TH, Ai B (2018) Metaphor representation and analysis of non-spatial data in map-like visualizations. ISPRS Int J Geo-Inf 7(6):225. 10.3390/ijgi7060225

[80] Lex A, Partl C, Kalkofen D, Streit M, Gratzl S, Wassermann AM et al (2013) Entourage: visualizing relationships between biological pathways using contextual subsets. IEEE Trans Visual Comput Graphics 19(12):2536–2545. 10.1109/TVCG.2013.154 10.1109/TVCG.2013.154 PMC4196276 24051820

[81] Nguyen HT, Bhatele A, Jain N, Kesavan SP, Bhatia H, Gamblin T et al (2021) Visualizing hierarchical performance profiles of parallel codes using CallFlow. IEEE Trans Visual Comput Graphics 27(4):2455–2468. 10.1109/TVCG.2019.2953746 10.1109/TVCG.2019.2953746 31751276

[82] Nobre C, Gehlenborg N, Coon H, Lex A (2019) Lineage: visualizing multivariate clinical data in genealogy graphs. IEEE Trans Visual Comput Graphics 25(3):1543–1558. 10.1109/TVCG.2018.2811488 10.1109/TVCG.2018.2811488 PMC6170727 29993603

[83] Chen SM, Chen S, Wang ZH, Liang J, Yuan XR, Cao N et al (2016) D-Map: visual analysis of ego-centric information diffusion patterns in social media. In: Proceedings of the IEEE conference on visual analytics science and technology (VAST), IEEE, Baltimore, 23–28 October 2016. 10.1109/VAST.2016.7883510

[84] Chen SM, Chen S, Lin LJ, Yuan XR, Liang J, Zhang XL (2017) E-Map: a visual analytics approach for exploring significant event evolutions in social media. In: Proceedings of the IEEE conference on visual analytics science and technology (VAST), IEEE, Phoenix, 3–6 October 2017. 10.1109/VAST.2017.8585638

[85] Chen S, Li SH, Chen SM, Yuan XR (2020) R-Map: a map metaphor for visualizing information reposting process in social media. IEEE Trans Visual Comput Graphics 26(1):1204–1214. 10.1109/TVCG.2019.2934263 10.1109/TVCG.2019.2934263 31425084

[86] Chan WY, Qu HM, Mak WH (2010) Visualizing the semantic structure in classical music works. IEEE Trans Visual Comput Graphics 16(1):161–173. 10.1109/TVCG.2009.63 10.1109/TVCG.2009.63 19910669

[87] Kumpf A, Tost B, Baumgart M, Riemer M, Westermann R, Rautenhaus M (2018) Visualizing confidence in cluster-based ensemble weather forecast analyses. IEEE Trans Visual Comput Graphics 24(1):109–119. 10.1109/TVCG.2017.2745178 10.1109/TVCG.2017.2745178 28866576

[88] De Luca F, Hossain I, Gray K, Kobourov S, Börner K (2019) Multi-level tree based approach for interactive graph visualization with semantic zoom. arXiv preprint arXiv: 1906.05996

[89] Xing YW, Dondi C, Borgo R, Abdul-Rahman A (2024) Visualizing historical book trade data: an iterative design study with close collaboration with domain experts. IEEE Trans Visual Comput Graphics 30(1):540–550. 10.1109/TVCG.2023.3326923 10.1109/TVCG.2023.3326923 37871084

[90] Sun GD, Wu YC, Liu SX, Peng TQ, Zhu JJH, Liang RH (2014) EvoRiver: visual analysis of topic coopetition on social media. IEEE Trans Visual Comput Graphics 20(12):1753–1762. 10.1109/TVCG.2014.2346919 10.1109/TVCG.2014.2346919 26356889

[91] Cui WW, Liu SX, Tan L, Shi CL, Song YQ, Gao ZK et al (2011) TextFlow: towards better understanding of evolving topics in text. IEEE Trans Visual Comput Graphics 17(12):2412–2421. 10.1109/TVCG.2011.239 10.1109/TVCG.2011.239 22034362

[92] Wang H, Lu YF, Shutters ST, Steptoe M, Wang F, Landis S et al (2019) A visual analytics framework for spatiotemporal trade network analysis. IEEE Trans Visual Comput Graphics 25(1):331–341. 10.1109/TVCG.2018.2864844 10.1109/TVCG.2018.2864844 30130225

[93] Wang JP, Liu XT, Shen HW, Lin G (2017) Multi-resolution climate ensemble parameter analysis with nested parallel coordinates plots. IEEE Trans Visual Comput Graphics 23(1):81–90. 10.1109/TVCG.2016.2598830 10.1109/TVCG.2016.2598830 27875136

[94] Wagner M, Slijepcevic D, Horsak B, Rind A, Zeppelzauer M, Aigner W (2019) Kavagait: knowledge-assisted visual analytics for clinical gait analysis. IEEE Trans Visual Comput Graphics 25(3):1528–1542. 10.1109/TVCG.2017.2785271 10.1109/TVCG.2017.2785271 29993807

[95] Tovanich N, Soulié N, Heulot N, Isenberg P (2022) MiningVis: visual analytics of the bitcoin mining economy. IEEE Trans Visual Comput Graphics 28(1):868–878. 10.1109/TVCG.2021.3114821 10.1109/TVCG.2021.3114821 34596542

[96] Wu YH, Deng DZ, Xie X, He MQ, Xu J, Zhang HZ et al (2023) Obtracker: visual analytics of off-ball movements in basketball. IEEE Trans Visual Comput Graphics 29(1):1–11. 10.1109/TVCG.2022.3209373 10.1109/TVCG.2022.3209373 36166529

[97] Deng ZK, Liu YB, Zhu MR, Xiang D, Yu HY, Su ZC et al (2025) TraSculptor: visual analytics for enhanced decision-making in road traffic planning. IEEE Trans Visual Comput Graphics 31(10):6899–6914. 10.1109/TVCG.2025.3532498 10.1109/TVCG.2025.3532498 40031195

[98] Wu YC, Lan J, Shu XH, Ji CY, Zhao KJ, Wang JC et al (2018) iTtvis: interactive visualization of table tennis data. IEEE Trans Visual Comput Graphics 24(1):709–718. 10.1109/TVCG.2017.2744218 10.1109/TVCG.2017.2744218 28866531

[99] Kwon BC, Choi MJ, Kim JT, Choi E, Kim YB, Kwon S et al (2019) RetainVis: visual analytics with interpretable and interactive recurrent neural networks on electronic medical records. IEEE Trans Visual Comput Graphics 25(1):299–309. 10.1109/TVCG.2018.2865027 10.1109/TVCG.2018.2865027 30136973

[100] Choi D, Drake B, Park H (2023) Co-embedding multi-type data for information fusion and visual analytics. In: Proceedings of the 26th International Conference on Information Fusion (FUSION), IEEE, Charleston, 27–30 June 2023. 10.23919/FUSION52260.2023.10224157

[101] Sacha D, Jentner W, Zhang LS, Stoffel F, Ellis G (2017) Visual comparative case analytics. In: Proceedings of the EuroVis workshop on visual analytics, Eurographics Association, Barcelona, 12–13 June 2017

[102] He JB, Wang XB, Wong KK, Huang XJ, Chen CJ, Chen ZX et al (2024) VideoPro: a visual analytics approach for interactive video programming. IEEE Trans Visual Comput Graphics 30(1):87–97. 10.1109/TVCG.2023.3326586 10.1109/TVCG.2023.3326586 37871060

[103] Zhao Y, Luo XB, Lin XR, Wang HR, Kui XY, Zhou FF et al (2020) Visual analytics for electromagnetic situation awareness in radio monitoring and management. IEEE Trans Visual Comput Graphics 26(1):590–600. 10.1109/TVCG.2019.2934655 10.1109/TVCG.2019.2934655 31443001

[104] Fujiwara T, Chou JK, Shilpika S, Xu PP, Ren L, Ma KL (2020) An incremental dimensionality reduction method for visualizing streaming multidimensional data. IEEE Trans Visual Comput Graphics 26(1):418–428. 10.1109/TVCG.2019.2934433 10.1109/TVCG.2019.2934433 31449024

[105] Robertson G, Fernandez R, Fisher D, Lee B, Stasko J (2008) Effectiveness of animation in trend visualization. IEEE Trans Visual Comput Graphics 14(6):1325–1332. 10.1109/TVCG.2008.125 10.1109/TVCG.2008.125 18988980

[106] Fuchs J, Fischer F, Mansmann F, Bertini E, Isenberg P (2013) Evaluation of alternative glyph designs for time series data in a small multiple setting. In: Proceedings of the SIGCHI conference on human factors in computing systems, ACM, Paris, 27 April– 2 May 2013. 10.1145/2470654.2466443

[107] Kim Y, Heer J (2021) Gemini: a grammar and recommender system for animated transitions in statistical graphics. IEEE Trans Visual Comput Graphics 27(2):485–494. 10.1109/TVCG.2020.3030360 10.1109/TVCG.2020.3030360 33079664

[108] Kim Y, Heer J (2021) : generating keyframe-oriented animated transitions between statistical graphics. In: Proceedings of the IEEE Visualization Conference (VIS), IEEE, New Orleans, 24–29 October 2021. 10.1109/VIS49827.2021.9623291

[109] Heer J, Robertson G (2007) Animated transitions in statistical data graphics. IEEE Trans Visual Comput Graphics 13(6):1240–1247. 10.1109/TVCG.2007.70539 10.1109/TVCG.2007.70539 17968070

[110] Chen Y, Du XM, Yuan XR (2017) Ordered small multiple treemaps for visualizing time-varying hierarchical pesticide residue data. Vis Comput 33(6–8):1073–1084. 10.1007/s00371-017-1373-x

[111] Han C, Jo J, Li AY, Lee B, Deussen O, Wang YH (2023) SizePairs: achieving stable and balanced temporal treemaps using hierarchical size-based pairing. IEEE Trans Visual Comput Graphics 29(1):193–202. 10.1109/TVCG.2022.3209450 10.1109/TVCG.2022.3209450 36166554

[112] Sondag M, Speckmann B, Verbeek K (2018) Stable treemaps via local moves. IEEE Trans Visual Comput Graphics 24(1):729–738. 10.1109/TVCG.2017.2745140 10.1109/TVCG.2017.2745140 28866573

[113] Mansmann F, Krstajic M, Fischer F, Bertini E (2012) StreamSqueeze: a dynamic stream visualization for monitoring of event data. In: Proceedings of the SPIE 8294, Visualization and Data Analysis 2012, SPIE, Burlingame, 22–26 January 2012. 10.1117/12.912372

[114] Hahn S, Trümper J, Moritz D, Döllner J (2014) Visualization of varying hierarchies by stable layout of voronoi treemaps. In: Proceedings of the 5th international conference on information visualization theory and applications (IVAPP), SciTePress, Lisbon, 5–8 January 2014. 10.5220/0004686200500058

[115] Tu Y, Shen HW (2007) Visualizing changes of hierarchical data using treemaps. IEEE Trans Visual Comput Graphics 13(6):1286–1293. 10.1109/TVCG.2007.70529 10.1109/TVCG.2007.70529 17968076

[116] Frishman Y, Tal A (2008) Online dynamic graph drawing. IEEE Trans Visual Comput Graphics 14(4):727–740. 10.1109/TVCG.2008.11 10.1109/TVCG.2008.11 18467750

[117] Zhou X, Liang X, Huang TL (2017) Time-varying complex network layout algorithm based on node centrality. Syst Eng Electron 39(10):2346–2352. 10.3969/j.issn.1001-506X.2017.10.28

[118] Gorochowski TE, Di Bernardo M, Grierson CS (2012) Using aging to visually uncover evolutionary processes on networks. IEEE Trans Visual Comput Graphics 18(8):1343–1352. 10.1109/TVCG.2011.142 10.1109/TVCG.2011.142 21860063

[119] Xu KS, Kliger M, Hero AO (2013) A regularized graph layout framework for dynamic network visualization. Data Min Knowl Discovery 27(1):84–116. 10.1007/s10618-012-0286-6

[120] Sheng SY, Chen ST, Dong XJ, Wu CY, Yuan XR (2021) Inverse markov process based constrained dynamic graph layout. J Comput Sci Technol 36(3):707–718. 10.1007/s11390-021-9910-5

[121] Che LM, Liang J, Yuan XR, Shen JP, Xu JQ, Li Y (2015) Laplacian-based dynamic graph visualization. In: Proceedings of the IEEE Pacific Visualization Symposium (PacificVis), IEEE, Hangzhou, 14–17 April 2015. 10.1109/PACIFICVIS.2015.7156358

[122] Archambault D, Purchase HC (2012) Mental map preservation helps user orientation in dynamic graphs. In: Proceedings of the 20th International Symposium on Graph Drawing, Springer, Redmond, 19–21 September 2012. 10.1007/978-3-642-36763-2_42

[123] Archambault D, Purchase HC (2013) The “map” in the mental map: experimental results in dynamic graph drawing. Int J Hum Comput Stud 71(11):1044–1055. 10.1016/j.ijhcs.2013.08.004

[124] Archambault D, Purchase HC (2012) The mental map and memorability in dynamic graphs. In: Proceedings of the IEEE Pacific Visualization Symposium, IEEE, Songdo, 28 February–2 March. 10.1109/PacificVis.2012.6183578

[125] Archambault D, Purchase H, Pinaud B (2011) Animation, small multiples, and the effect of mental map preservation in dynamic graphs. IEEE Trans Visual Comput Graphics 17(4):539–552. 10.1109/TVCG.2010.78 10.1109/TVCG.2010.78 20498503

[126] Marriott K, Purchase H, Wybrow M, Goncu C (2012) Memorability of visual features in network diagrams. IEEE Trans Visual Comput Graphics 18(12):2477–2485. 10.1109/TVCG.2012.245 10.1109/TVCG.2012.245 26357156

[127] Lin CC, Lee YY, Yen HC (2011) Mental map preserving graph drawing using simulated annealing. Inf Sci 181(19):4253–4272. 10.1016/j.ins.2011.06.005

[128] Zhao X, Fu SW, Yang R, Yang L, Chen YP, Zhang J et al (2025) Investigating visual perception of degree centrality in graph visualization. IEEE Trans Visual Comput Graphics 31(6):3679–3692. 10.1109/TVCG.2025.3567129 10.1109/TVCG.2025.3567129 40354207

[129] Fruchterman TMJ, Reingold EM (1991) Graph drawing by force-directed placement. Softw Pract Exp 21(11):1129–1164. 10.1002/spe.4380211102

[130] Kamada T, Kawai S (1989) An algorithm for drawing general undirected graphs. Inf Process Lett 31(1):7–15. 10.1016/0020-0190(89)90102-6

[131] Zhong FH, Xue ML, Zhang J, Zhang F, Ban R, Deussen O et al (2024) Force-directed graph layouts revisited: a new force based on the t-distribution. IEEE Trans Visual Comput Graphics 30(7):3650–3663. 10.1109/TVCG.2023.3238821 10.1109/TVCG.2023.3238821 37021999

[132] Xue ML, Wang Z, Zhong FH, Wang Y, Xu ML, Deussen O et al (2023) Taurus: towards a unified force representation and universal solver for graph layout. IEEE Trans Visual Comput Graphics 29(1):886–895. 10.1109/TVCG.2022.3209371 10.1109/TVCG.2022.3209371 36166546

[133] Noack A (2003) An energy model for visual graph clustering. In: Proceedings of the 11th International Symposium on Graph Drawing, Springer, Perugia, 21–24 September 2003. 10.1007/978-3-540-24595-7_40

[134] Jacomy M, Venturini T, Heymann S, Bastian M (2014) ForceAtlas2, a continuous graph layout algorithm for handy network visualization designed for the Gephi software. PLoS One 9(6)e98679. 10.1371/journal.pone.0098679 10.1371/journal.pone.0098679 PMC4051631 24914678

[135] Gansner ER, Koren Y, North SC (2004) Graph drawing by stress majorization. In: Proceedings of the 12th International Symposium on Graph Drawing, Springer, New York, 29 September–2 October 2004. 10.1007/978-3-540-31843-9_25

[136] Hu YF (2006) Efficient, high-quality force-directed graph drawing. Math J 10(1):37–71

[137] Fan YL, Lyu X, Wang L, Zhao Y, Zhou FF, Wang Y (2026) How well will LLMs perform for graph layout tasks? Visual Inf 10(1):100285. 10.1016/j.visinf.2025.100285

[138] Wang YY, Bai ZN, Lin ZF, Dong XQ, Feng YCJ, Pan JC et al (2021) G6: a web-based library for graph visualization. Visual Inf 5(4):49–55. 10.1016/j.visinf.2021.12.003

[139] Sun MD, Cai LG, Cui WW, Wu YQ, Shi Y, Cao N (2023) Erato: cooperative data story editing via fact interpolation. IEEE Trans Visual Comput Graphics 29(1):983–993. 10.1109/TVCG.2022.3209428 10.1109/TVCG.2022.3209428 36155449

[140] Danneman N, Gove R (2022) Tuning automatic summarization for incident report visualization. In: Proceedings of the IEEE 15th Pacific Visualization Symposium (PacificVis), IEEE, Tsukuba, 11–14 April 2022. 10.1109/PacificVis53943.2022.00031

[141] Zhu SJ, Sun GD, Jiang Q, Zha M, Liang RH (2020) A survey on automatic infographics and visualization recommendations. Visual Inf 4(3):24–40. 10.1016/j.visinf.2020.07.002

